# Speaking with care: a qualitative study on communication styles in everyday nursing conversations with older persons

**DOI:** 10.1186/s12877-025-06819-3

**Published:** 2025-12-05

**Authors:** Annelie J. Sundler, Jessica Höglander, Tanja Gustafsson, Inger K. Holmström

**Affiliations:** 1 Faculty of Caring Science, Work Life and Social Welfare, University of Borås, Borås, SE-501 90 Sweden; 2 School of Health, Care and Social Welfare, Mälardalen University, Västerås, Sweden; 3 Department of Public Health and Caring Sciences, Uppsala University, Uppsala, Sweden; 4 The Swedish Red Cross University, Stockholm, Sweden

**Keywords:** Communication, Deductive content analysis, Home care, Nursing, Nurse assistants, Older adults, Older people, Person-centred care

## Abstract

**Background:**

Enhancing communication in everyday nursing conversations is essential for promoting person-centred care and preserving the dignity of older persons. This qualitative study was performed to gain an in-depth understanding of the nursing assistants’ person-centred communication skills and their communication style. The aim was to explore the communication styles of nursing assistants during home care visits to older persons.

**Methods:**

This deductive qualitative study was based on observational data gathered with audio recordings of naturally occurring communication between nursing assistants and older persons during home care visits. Data collection was part of an educational intervention study for in-home nursing assistants and took place from March to December 2022. Data were analysed with a method for deductive qualitative content analysis. The deductive analysis was guided by the framework developed by Bottorff and Morse, and the communication styles identified in the current study, *doing more*,* doing with*,* doing for*,* and doing tasks.*

**Findings:**

A total of 70 audio recording home care visits, including 39 nursing assistants and 37 older persons were included. Based on conversations during these visits, four distinct communication styles employed by NAs in their everyday interactions with older persons were described in depth with focus on communication patterns observed. The different styles included nuances of communication ranging from being more relational and collaborative to more task-oriented interactions. A pattern of *doing too much* was also observed.

**Conclusions:**

The communication styles used influenced the degree of person-centredness. Nurse assistants’ communication styles can vary during conversations and appear to reflect individual traits. The emotional demands of home care work may require more recognition and support from policymakers and managers. Strengthening person-centred communication and care for older persons requires both training and organizational support.

## Background

Enhancing communication in everyday nursing conversations is essential for addressing the health and well-being of patients [1]. Home care involves providing healthcare or assistance in a patient’s home, where caring for older persons extends beyond performing tasks to building relationships based on commitment and support. Nurses and nursing assistants (NAs) play crucial roles in assisting individuals living with frailty or chronic illness, offering not only physical care but also emotional and existential support. This includes addressing concerns related to aging and illness, acknowledging emotions tied to lived experiences, and affirming individuals’ perspectives [2]. Home care services are designed to enable older persons to live at home for as long as possible [3].

This study was conducted in Sweden as part of a project focused on person-centred communication for NAs working in home care services [3]. NAs provide most of the direct care to older adults in Sweden and constitute one of the country’s largest occupational groups [4], with approximately 135,000 employed in municipal home care or nursing homes. However, increasing workload and job complexity have made it challenging to retain skilled staff and maintain supportive working conditions [4, 5].

The complexity of providing in-home nursing care for older persons presents numerous challenges for NAs, particularly with respect to communication and ethical and existential concerns [2, 6–8]. In addition, organizational structures, such as regulations, time limits, efficiency and more standardized care [2, 7, 9], further add to these challenges and restrict NAs’ control over their daily work.

A relatively large proportion of municipal care employees in Nordic countries are reported to have low qualifications, with some lacking the necessary competencies to fully meet the care needs of older persons [10, 11]. In Sweden, most NAs have vocational training from high school health education programs, and the workforce is predominantly female (91%). While the number of untrained employees is estimated to be lower than the number of those with formal training, challenges remain in ensuring the appropriate competencies among staff in municipal care for older persons, such as competence in person-centred care (PCC) and communication. Additionally, over one-third of nursing home staff are born abroad [12]. The Health and Social Care Inspectorate in Sweden reported that 97% of municipalities employ staff who lack sufficient language skills, which can make the communication chain fragile [13]. Furthermore, NAs play an important role in reporting changes in older persons’ health status to a registered nurse, who in turn contacts a physician if necessary. Effective communication is therefore essential not only for patient care but also for teamwork and collaboration among staff to ensure that care is tailored to the needs and health status of older persons.

### Person-centred care and communication

The PCC places the person at the centre of health and care processes, emphasizing a compassionate care approach with empathy, respect and active engagement where relational aspects and communication are essential [14]. This person-centred approach is especially relevant for NAs in home care, as it involves recognizing and supporting dignity and a meaningful life for older persons. Moreover, NAs providing home care can play a crucial role in fostering autonomy and well-being. Such care needs to be grounded in respectful and dignified communication that acknowledges individual needs and experiences. Previous research has suggested that communication and PCC are particularly helpful in preserving dignity, empathy, well-being, and humanity in the care of older persons [2, 15, 16].

PCC may also increase job satisfaction for NAs [17]. Thus, person-centred communication skills are essential for providing individualized nursing care to older persons. Bottorff and Morse [18] identified four patterns of attending to patients’ needs. The four patterns were originally developed in a cancer care context but have subsequently also been used in other contexts. For example, Wadensten [19] used this approach when investigating morning conversations in nursing homes. The four patterns are labelled *doing more*,* doing with*,* doing for*,* and doing tasks* [18]. *Doing more* includes “doing something” beyond what is usually required to complete care. It is characterized by an engaged relationship. *Doing with* is characterized by an equal focus on the task and the patient. This type of attending implies a willingness to work cooperatively with patients. In *doing for*, the primary focus is on responding to patient requests and needs that are not related to treatment. It is characterized by a personalized approach to providing assistance and often involves “extras”. Finally, *doing tasks* is characterized by an indifferent, apathetic, or routinized approach, with a focus on equipment, treatment, and getting the job done [18]. The attending pattern of *doing more* seems to be rather similar to PCC, with the engaged relationship being central.

While these attending patterns have been examined in clinical and institutional healthcare settings, little is known about how they are expressed during ordinary home care visits, particularly in the communication between NAs and older persons. Given that communication is central to person-centred care, exploring these patterns with a specific focus on communicative behaviours in NA–older person interactions can offer valuable insights into how care relationships are formed and sustained in the home care context. Caring for older persons often involves addressing emotional needs that may be subtly expressed and easily overlooked [2, 6]; person-centred communication requires recognising, inviting, and involving older persons, with the caregiver’s attentiveness and responsiveness playing a crucial role. However, research on NAs’ communication styles and how these may reflect different approaches and underlying perspectives in home care remains limited. Given the potential influence of communication on care quality and person-centred practice, an in-depth, deductive analysis of attending patterns with an emphasis on communication skills can contribute important knowledge to guide both practice and training for NAs.

### Aim

This study aims to explore the communication styles of NAs during home care visits with older persons.

## Methods

### Design

A qualitative design was used to explore the communication style of NAs in conversations with older persons during home care visits. The data collection was part of a larger study, A person‑centred CommunicaTION (ACTION) project, and the NAs who participated in this study were offered an educational intervention [3]. Data was collected with observations using audio recordings of naturally occurring communication between NAs and older persons during home care visits. As the primary data consisted of observations with audio recordings during home care visits this study follows the STROBE guidelines [20] for the conduct and dissemination of observational studies. Although the recordings were made while the intervention was ongoing, they were not intended to assess its outcomes. Rather, they served to deepen our understanding of communicative practices in the context of home care.

### Setting and sample

The study was conducted in seven home care units in mid-Sweden, located in both urban and rural areas, to capture variation in organisational and contextual conditions. Purposive sampling was used to recruit NAs within these units to ensure diversity in the data while remaining within the study’s scope. All participants were recruited from home care units whose managers had agreed to implement the educational intervention. Within these units, only NAs with ongoing, non-casual employment (i.e., excluding temporary hourly staff) were invited to participate in the study. One of the researchers visited all participating units to provide information about the research process and the educational intervention, and to invite eligible NAs to participate.

The resulting sample included NAs with diverse background characteristics, reflecting variation in years of experience, age, and gender, as well as differences in work schedules (day/evening) and the types of care situations they typically encountered. Dropouts or missing audio recordings occurred because of factors such as high workload, feelings of insecurity or discomfort with recording, or simply forgetting to bring the audio recorder to visits.

### Data collection

The observational data were collected via audio recordings of home care visits to capture the communication between NAs and older persons during these visits. As part of a larger study, recordings were collected from March 2022 until December 2022, during an educational programme. The NAs were responsible for managing the recordings and were provided with instructions on how to operate the equipment and handle situations where an older person might feel uncomfortable or hesitant about being recorded.

A portable, user-friendly recording device was chosen for convenience. The NAs recording the visits were instructed to start the audio recording when they entered the older person’s home and ended it when they left. They were also instructed to inform the older person they were visiting about the recording when entering their home. The recording equipment was placed either on the upper arm of the NAs or on a visible surface somewhere near the nurse and the older person.

### Data analysis

A deductive qualitative content analysis [21] was conducted. This approach allows data to be analysed via a predefined set of categories derived from research or the literature. With this approach, the researchers used an established set of categories as a guide for coding and interpreting the data to expand existing knowledge while remaining open to new insights that may emerge during the analysis. The data were coded directly from audio recordings without being transcribed.

In the present study, deductive analysis was guided by the preexisting behavioural patterns identified by Bottorff and Morse [18]: *doing more*,* doing with*,* doing for*, and *doing tasks*. These attending patterns were used to explore different communication styles employed by NAs in their everyday interactions with older persons. These predefined categories served as a framework for analysing daily nursing conversations, with the interactions between older persons and NAs treated as the structural units of analysis. The analysis specifically focused on differences between the styles and communication skills used, including characteristics and nuances of the communication. The authors attempted to delve deeper into the data, reflecting on communication details, to allow new insights to emerge. From this process, communication patterns observed were elaborated through a reflective process.

The deductive content analysis [21] was carried out in three phases: preparation, organizing and reporting. In the preparation phase, two of the researchers listening to all the audio recordings in full to gain an overview and a general understanding of the data. Each recording was then listened to attentively to explore the NAs’ communication styles, with detailed notes taken on verbal and non-verbal cues, including intonation, pauses, and other interactional subtleties. Time stamps were recorded for segments containing notable communicative events or behaviours, enabling their retrieval and closer examination during subsequent stages of the analysis. Observations were continuously discussed among the researchers and systematically noted, linking episodes to specific time points of the recordings. Notes were made from all the recordings, and significant sequences were selected for further in-depth analysis of meaning.

The organizing phase included developing a structured analysis matrix in which communication styles were labelled according to the framework of Bottorff and Morse [18]. The data were then coded into this matrix and critically examined to refine and elaborate the categories, with particular attention to the communication patterns employed by NAs. According to Elo and Kyngäs [21], it is possible to choose either only the aspects from the data that fit the categorization frame or, alternatively, those that do not fit as well. In this study we used all previously labelled attending patterns of Bottorff and Morse [18] and further refined and developed these with focus on communication skills in the home care context.

The iterative analysis involved researchers revisiting the steps repeatedly to validate, revise, and refine the findings [22]. This deductive approach enabled a nuanced examination of how previously described attending patterns were manifested within the NAs communication with older persons in the specific context of home care. Throughout the analysis process, recordings were paused for discussions on communication nuances, message details, and observed meanings. This approach is particularly important in communication research, as meaning is conveyed not only through spoken words but also through intonation and context. Finally, the findings are described in the results section below, illustrated with excerpts from conversations between older persons (OPs) and nurse assistants (NAs), with each encounter assigned a number from 1 to 70.

## Findings

### Sample description of observed audio recorded home care visits

A total of 70 audio recorded visits (ARV), involving 39 nursing assistants and 37 older persons, were included. The duration of the visits ranged from 1 to 56 min (Mean = 17 min), measured from the start to the end of each recording. For an overview of participant characteristics see Table 1. Each NA recorded between 1 and 4 visits. Each older person participated in 1 or more recordings, up to a maximum of 6, with one or more of the NAs.

**Table 1:** Characteristics of the NAs and older persons who participated in the audio recordings

	NAs (*n* = 39)	Older persons (*n* = 37)
**Characteristics of the participants**		
Sex		
Female n (%)	36 (92)	24 (65)
Male n (%)	3 (8)	13 (35)
Age, mean (range)	43 (26–62)	84 (68–103)

### Communication styles in everyday NA conversations with older persons

The communication styles observed were structured based on the four patterns of attentiveness in nursing care described earlier —*Doing more*, *Doing for*, *Doing with*, and *Doing tasks* [18]. These patterns illustrate a spectrum of communication styles among NAs, ranging from relational and collaborative to more task-oriented interactions. A difference between styles was how the use of communication skills could shift the focus of care from mere task completion to include emotional and relational aspects. NAs appear to navigate between offering emotional and individualized support and focusing on practical tasks during conversations. Communication styles could vary both between visits and within the same visit, and seemed to be influenced by individual traits, as observed from NAs with multiple recordings. For an overview the communication styles and their characteristics see Table 2.

**Table 2:** An overview of the characteristics and nuances of the four communication styles in everyday NAs conversations – from relational to task-oriented

	Doing more	Doing for	Doing with	Doing tasks
**Focus**	Going beyond immediate tasks by actively anticipating needs and providing emotional support	Providing care and tasks on behalf of the care recipient and ensuring their comfort	Encouraging participation and collaboration on care and tasks	Prioritize efficiency and task completion
**Balance between emotional and practical support**	High emotional support, moderate practical involvement	Moderate emotional support, low practical involvement	Moderate emotional support, high practical involvement	Low emotional support, low practical involvement
**Nature of conversations**	A person-centred approach that actively involves and emotionally engages with the individual	Supportive care delivered for the individual, potentially reducing their role to that of a passive recipient	Encouraging and cooperative to involve the person in their care	Functional and task-oriented to get job done
**Interaction style**	Relational, responsive and comforting	Supportive	Collaborative and comforting	Distanced with limited attention beyond tasks
**Tone of voice**	Enthusiasm and warm tone	Polite and gentle tone	Respectful and gentle tone	Polite and gentle tone but non-individualized
**Facilitative communication behaviours**	Active listening, emotional affirmation and providing reassurance	Reassurance and provide space for individual concerns	Offering choices and positive reinforcement	Provides clear instructions and checks for understanding, with minimal personal conversations

Overall, conversations during the home care visits predominantly consisted of everyday talk related to social interaction with the older person. These conversations were commonly focused on topics such as daily routines or shared interests, emphasizing social aspects of caregiving. However, there were relatively few instances of challenging or emotionally complex interactions, which may reflect either the nature of the visits or the NAs’ comfort level in addressing more sensitive topics.

#### Doing more

Doing more was characterized by daily conversations in which the NAs went beyond the immediate tasks to be performed. These conversations often involved NAs actively anticipating an older person’s needs and providing emotional support or encouragement. For example, NAs could actively engage with older person by addressing individual needs or offering comfort during times of distress or in daily conversations. An excerpt from a visit with an older male, conducted by an NA, is provided below. They share a common interest in hunting, and much of their conversation revolves around this topic (ARV 55):

NA: Have you been doing OK recently?

OP: Well, yes, but it was great to hear ‘bout the moose-hunting team ….

NA: Yes, they’re out now … I’ve been there a few times myself. It’s rather exciting to be around out in the woods.

OP: Yeah, it truly is!

NA: [*Keeps talking about the current hunt*]

OP: [*laughing*]*…* Then, you can cook the moose’s tongue as well.

NA: If we want to, I’m not sure we will ….

OP: I did when I had been hunting. [*Describe how the tongue should be cooked and prepared*].

NA: Yes, yes…can you please lift your foot up a bit? Does it hurt at all?

OP: No, it’s just itching ….

NA: Am I tickling you?/*/Pause//* Are you OK even though your legs are somewhat hurting?

OP: It’s when I should try to stand up that they hurt like hell ….

This conversation illustrates a meaningful exchange that seems to enhance the emotional well-being of the older person. In the conversation, they seem to have common ground, where they know each other well, and their communication shifts seemingly effortlessly between small talk and discussions of care and health issues.

Another example is from a visit where the NA is ‘doing more’ than expected. In this interaction, the NA goes beyond the planned morning care by combining physical care with relational engagement. The NA engages the older person in friendly, personalised conversation that is appreciated by the older person who contrasts this attentiveness with the less consistent practices of other staff (ARV 11).

NA: I’m here to assist you with some morning care…did you sleep well [name of the older person]?

OP: Yes, yes. I always sleep well.

NA: Great, that’s great. Me too, I slept well. Now I’ll put some ointment on….

OP: Oh, that’s nice. I can feel you’ve been doing this before, you’re so skilled.

NA: Thanks, I do this to everyone.

OP: The others, the other NAs, they often forget….

NA: But skincare is important.

OP. And my skin are so dry these days….

NA: Shall we change your nightie as well? It seems sweaty.

OP: Yes, thanks…it’s a bit itchy. But I don’t think it’s stained in any way….

NA: No, no, but your back is itchy, is it? Then I’ll put some ointment on your back and grease you up as well. Okay?

OP: Okay, that’s feels so good, so good….

This ‘doing more’ thus encompasses both the physical extension of care tasks and the provision of emotional value.

Within this pattern, however, a form of “doing too much” was also noted. This was apparent, for instance, in the present excerpt (ARV 23):

NA: Well, how was your day today?

OP: It’s been a good day, I’d say.

NA: That’s great, wonderful!

OP: I’m up and about… [*goes on to describe the day in detail*] watch the telly.

NA: Wow!!!

OP: … Talking to a neighbour, having dinner, drinking some coffee….

NA: [*laughing loudly*] *…* You’ve done SOO much! That’s GREAT! Is it a neighbour you usually talk to, or do you just greet each other normally?

OP: I’ve lived here for some 30 years, so I know the people living here….

NA: Yes, it’s so FANTASTIC when you can talk and greet and so on!

OP: Oh, yes, yes.

NA: Yes, yes … you mentioned dinner, what kind of good food did you have today?

The excerpt illustrates how patterns of doing more, when overdone, can appear unnatural or exaggerated, undermining the genuine warmth and authenticity of the interaction. Even though the NA shows interest in the older person’s life and daily activities by asking questions, she fails to attune to the older person’s state of mind. As a result, the communication style shifts from ‘doing more’ to ‘doing too much.’ Despite the use of a cheerful tone and positive expressions such as ‘wow,’ ‘great,’ ‘fantastic,’ and ‘wonderful,’ along with frequent laughter, it has become overwhelming and inauthentic.

In the ‘doing more’ style, NAs generally balance high emotional support with moderate practical involvement while remaining responsive to individuals’ views and needs and validating the older person’s perspective.

#### Doing for

This communication style was characterized by conversations and actions in which the NAs provided care and performed tasks on behalf of the care recipient, ensuring their comfort.

The NAs assumed responsibility for tasks and decision-making for the older adult. While supportive and caring, this style sometimes appeared to limit the older person’s opportunities to actively participate in their care or express their preferences. This is illustrated in the following excerpt (ARV 69):

NA: What do you do when you are at the daycare centre? Are you talking together?

OP: Not much, we’re ….

NA: Are there special activities arranged?

OP: We eat … but I’m only there for like two hours ….

NA: Well, then there’s limited time to do something special. However, it’s good to see some people and chat a bit.

OP: Umm … staff and ….

NA: Well, it’s nice for you to come out see some other people and ….

In this example, the NA engages in small talk and asks the older person about their experiences at the day-care centre but provides limited opportunity for the older person to truly answer. Instead, the NA interrupts and puts words in the older person’s mouth, although the person is encountering more as a passive recipient where the NA asks but does not listen and instead speaks on their behalf. Thus, the person is engaged more as a passive recipient, and the NA controls the conversation about their day, for instance, when the NA asserts how nice it is to meet other people at the day-care centre.

This style was observed in conversations where the NAs provided moderate emotional support, with a low degree of involvement from the older person in either the conversation or the practical tasks being performed. In these instances, NAs typically help by doing things for the person. While the NAs appeared supportive, polite, and gentle, the older person’s participation remained minimal.

#### Doing with

This collaborative style was characterized by NAs actively encouraging OPs participation in conversations about care and tasks. The NAs appeared to work together with the older person. By using a respectful and gentle tone, they offered choices and fostered a sense of partnership through the way they communicated with the person.

Below is an example of communication (ARV 55) during care activities, where the NAs help with putting on support stockings:

OP: That feels truly nice.

NA: Does it feel good on the foot?

OP: Super!

NA: Right … now we’re done with that one. Let us take the other support stockings now.

*Pause*

OP: You need to be a pro to put these on cause my daughter tried to help me the other day and she didn’t truly know how to do it properly …

This example briefly illustrates a collaborative approach, where the NA and the older person work together on the task at hand. The NA is reflective and checks in with the person’s perspective while putting on stockings. Throughout the interaction, it appears that they are collaborating on the task, with the NA kindly ensuring that the older person is comfortable.

‘Doing with’ also involves conversations where NAs encourage the capacity of the older person for own decisions. The style involves a collaborative approach where the NAs use a friendly and respectful tone of voice. This can be illustrated in a situation where an NA is supportive when the older person during a visit is talking about moving to new housing. The NA listens attentively, acknowledges the OP’s feelings about being a burden for relatives, and explicitly affirms her capacity to manage independently. The NA says: ‘Oh yes, of course you want to manage on your own. And you do! You are a fighter.’ (ARV 41). Such affirmations positioned the OP as capable and resilient, reframing the decision as an expression of independence rather than dependency.

During conversations, NAs also encourage older persons’ involvement in care, tasks and everyday life decision, characterized by high involvement from OPs, accompanied by support and positive reinforcement. They typically use a positive and gentle tone when offering choices, inviting the OP to be as active as possible. This style often conveys the following message: ‘We are doing this together’.

#### Doing tasks

Doing tasks is a functional, task-oriented communication style where the priority is to complete tasks efficiently and on time. This approach was observed in activities such as serving meals or assisting with personal hygiene, with minimal social or emotional engagement with the older person. The communication style appears to be partly driven by organizational factors, such as time constraints for each task, with NAs expressing pressure to complete their duties in time for the next home visit.

The following example illustrates a conversation focused on the medical task at hand (ARV 07):

NA: Today, you’re going to take your medications.

OP: Hmm, yes, I know.

NA: You knew about your pills.

OP: Pill, it’s only one pill.

NA: Yes, it’s only one pill.

OP: That’s not too hard to take.

NA: However, you have no shower planned today.

These brief conversations are centred around the NA’s task at hand: administering medication and commenting that the older person should not shower. It is unclear whether the older person intended to shower, but the mention of it disrupted the brief exchange of medication. The NA provides instructions with minimal verbal interaction and limited engagement beyond the task. While the communication is functional, it does not create space for further discussion about the medication or any other thoughts or concerns the older person may have.

In another excerpt below (ARV 62) the older person brings up a sensitive topic, but the NA shows no interest in discussing it:

OP: Today, they will pay me a visit to assess my memory ….

NA: Uhu… why?

OP: Well, I’ve asked to … to get assessed to know if I truly have an organic memory-loss, that is, an increasing loss of memory of organic nature, or if I in fact am … hmmm … let’s say depressed, to use that word.

NA: OK.

OP: And they’ll visit me from the team, it’s a nurse and a psychologist … I think.

NA: OK.

OP: And they provide an assessment form containing 16 pages with questions.

When the older person first mentions the memory assessment, the NA responds with a brief “Why?” but shows no further interest in the answer or the older person’s state of mind. Background sounds suggest that she is engaged in other chores rather than focusing on the conversation. Later, in the nearly 30-minute encounter, the older person brought up memory problems again, indicating that the topic was of concern to her. However, the NA once more responds in an absent-minded manner. Doing tasks was observed as a communication style that was functional, brief and task oriented.

## Discussion

The findings describe various communication styles used in conversations with older persons during home care visits. While the communication style of NAs may shift during a visit, their communication style primarily appears to reflect a personal trait, as observed from NAs participating in two or more recordings.

The exploration of communication styles in this study provides valuable insights into NA-older person interactions and the relational aspects of the care delivered. Communication patterns illustrate opportunities to enhance the relational and emotional aspects of caregiving while balancing efficiency and personalization. However, a key distinction between the patterns seems to reflect the differences in the underlying perspectives and goals of person-centred versus patient-centred care. A review of reviews on person-centred and patient-centred care highlights the differences between these concepts [14]. While the primary goal of patient-centred care is to achieve a functional life by addressing medical needs and optimizing physical health, the PCC emphasizes supporting a meaningful life by acknowledging individual experiences, values, and personal perspectives. This distinction highlights the shift from solely addressing health conditions to fostering a sense of meaning and well-being in everyday life. The nature of the interpersonal interaction and communication needed to succeed with care actions in home care and support older persons’ dignity and emotional concerns can be challenging [2]. Moreover, addressing emotional concerns may be uncomfortable and requires NAs to have courage, as well as training [23]. Relational aspects may be important for both care recipients and NAs. A recent study highlighted that relationships with clients and the emotional aspects of care work are central to workers’ perceptions of well-being and work-life balance [24]. Successfully managing various care situations appears to depend on the individual NA’s communication skills. Moreover, the current study illustrates how person-centred communication is linked to more individualized care, supporting well-being and a sense of meaning in everyday conversations with older persons in home care, aspects that are inherent to the relational nature of care work.

The communication style of the NAs influenced the care provided to the older persons in different ways. Depending on the communication behaviours used, as illustrated in Table 2, the NAs’ communication styles varied in terms of their degree of person-centredness. Communication, respect, and empathy are well known as central to the PCC [14], and the ability to listen is imperative for acknowledging and validating emotions, which are key to person-centredness [25]. Hence, emotional communication plays a central role in the PCC and the development of trusting relationships. Furthermore, the findings offer insights into the significance of both the spoken words and the relational aspects of engaging with older adults and recognizing emotional dimensions, important communication factors linked to the level of person-centredness.

The styles observed in this study ranged from highly relational, i.e., *doing more* and *doing with*, to more task-oriented, i.e., *doing for* and *doing tasks*. NAs who engaged in d*oing more* demonstrated proactive engagement, emotional affirmation, and responsiveness to the older person’s needs. While this style fostered relational depth, a phenomenon of “doing too much” was noted in the present data, where NAs’ enthusiasm risked overshadowing the older person’s contributions, which has not been described by Bottorff and Morse [18] or Wadensten [19]. This suggests that while emotional support is a key element of person-centred communication, excessive affirmation may lead to inauthentic or overwhelming interactions.

Conversely, *doing for* reflected the communication behaviours of the NAs, who performed care tasks on behalf of the older persons, with the underlying goal of focusing on the older person’s functional life and ensuring task completion. However, this approach limits opportunities for older person to actively participate in their care. Gjerberg et al. [26] reported that while many nursing home patients and their relatives trusted staff to make decisions, this often led to a passive role for patients. This aligns with the *doing for* approach, where reliance on staff can limit the older person’s autonomy. Gjerberg et al. [26] emphasized the need to initiate conversations about care preferences to ensure alignment with patients’ values. The incorporation of shared decision-making can balance task completion with patient involvement, enhancing the relational aspects of care and supporting autonomy.

Similarly, *doing tasks* was characterized primarily by efficiency-driven, functional communication with minimal personal engagement. Notably, within this task-oriented style, some NAs missed opportunities to respond to emotional cues from older persons, particularly in relation to sensitive topics. This aligns with previous research indicating that time constraints and task prioritization may not always allow NAs to respond to and acknowledge older persons’ concerns [2], which can also serve as barriers to implementing PCC in practice [27]. Having person-centred communication skills, shared decision-making capabilities, motivation, and respect for older persons’ autonomy are suggested cornerstones in the development towards a more effective implementation of PCC [27].

The different communication styles also reflect various degrees of NAs’ attentiveness to verbal and non-verbal cues. For example, *doing with* appeared to involve communication behaviours that employed more facilitative communication strategies, such as inviting older persons to express their preferences and providing reassurance. As noted by Wadensten [19] in a nursing home context, staff need to be aware of their important role because they set the stage for communication in home care encounters. Emotional conversations are also context-bound, meaning that the specific tasks being carried out during visits influence the worries expressed [28]. As a result, NAs need a high level of situational sensitivity and emotional attunement to respond effectively. Further studies are needed to explore older person’ opinions of how good communication can be achieved.

### Strengths and limitations

A strength of this study is its observational design using audio recordings, as such data can provide valuable insights into interactions and actual communication [29]. Audio recordings are suitable for capturing real-life conditions, such as home care visits, in their natural context. Compared with self-reported data, such as interviews or surveys, audio recorded observations have the advantage of counteracting gaps between what participants report and what they remember about a phenomenon [29]. It offers more direct insight into how communication unfolds in clinical practice. Although this design does not include participants’ self-reported perspectives, we argue that the use of naturalistic observations strengthens the study credibility by providing direct access to authentic interactional data.

Compared with video recordings, audio recordings are more limited in capturing the nuances of non-verbal communication. While audio captures tone of voice, pacing, and responsiveness, video also includes facial expressions, gestures, and body movements. However, previous research has reported a high level of agreement in emotional tone ratings between audio and video data [30], and video recordings did not yield results significantly different from audio alone, despite offering additional non-verbal cues. Thus, audio recordings can still effectively capture affective messages in both verbal and non-verbal aspects of communication, enriching the understanding of emotional and relational dynamics.

Additionally, the presence of a recording device may influence NAs’ communication behaviours, potentially resulting in more deliberate or structured interactions. However, earlier research also suggested that this effect is generally limited, particularly when recordings occur in familiar care environments and without researchers present [30].

Two researchers participated in all phases of the analysis, engaging in regular discussions to critically examine interpretations and enhance trustworthiness. Both are registered nurses and professors with extensive prior research experience in home care and communication. We remained aware of these backgrounds throughout the analysis and engaged in critical reflection to minimise potential bias, aiming instead to use our expertise to illuminate important nuances in the data.

## Conclusions

This study contributes to the growing body of research on communication in home care by offering an in-depth exploration of NAs’ communication styles in daily conversations with older persons during home care and how these styles influence the degree of person-centredness. It shows how different communication styles can shift the focus of care from task completion to include emotional and relational aspects. Although these styles varied during home care visits, they appeared to be influenced by individual traits.

Relational aspects and communication skills, such as listening, responsiveness, and emotional engagement, were found to be linked to more individualized care. However, even well-intended communication can become overwhelming if it is excessive, potentially overshadowing the older person’s voice. While a task-oriented approach may be efficient, it can also result in missed opportunities to respond to emotional cues, underscoring the need for situational awareness and emotional attunement.

Although this study’s findings show that NAs may shift between communication styles within and across visits, determining when a particular style is most appropriate requires further investigation. The results suggest that flexibility in using different styles, rather than strict adherence to a single style, may be central to effective, person-centred communication in home care. Future research should explore how communication styles are linked to other factors in the working environment and their appropriateness in different situations.

Supporting NAs in developing communication skills, especially for emotional and person-centred conversations, may enhance the quality of care. Moving forward, strengthening person-centred communication in home care may require not only training but also structural and organizational support to manage emotional demands and facilitate meaningful interactions between NAs and older persons.

## Data Availability

The data that support the findings of this study are not openly available due to the sensitive nature of the data and ethical considerations. The data are located in controlled access data storage at the University of Borås and can be available from the authors upon reasonable request.

## Abbreviations

ACTION: A person‑centred CommunicaTION project
ARV: Audio recorded visit
NAs: Nursing assistants
OP: Older person
PCC: Person-centred care
